# Reproducibility of a Shear Wave Elastography Procedure for Assessing the Piriformis Muscle Stiffness in a Sample of Healthy Young Adults Under Controlled Laboratory Conditions: An Intra- and Inter-Examiner Reliability Study

**DOI:** 10.3390/jcm15124548

**Published:** 2026-06-11

**Authors:** Umut Varol, Mateusz D. Kobylarz, Mónica López-Redondo, Davinia Vicente-Campos, Sandra Sánchez-Jorge, Jorge Buffet-García, Juan Antonio Valera-Calero

**Affiliations:** 1 Grupo de Investigación de Alto Rendimiento en Evaluación Multidimensional y Tratamiento del Dolor Crónico, Universidad Rey Juan Carlos, 28922 Alcorcón, Spain; umut.varol@alumni.ie.edu; 2 Akademia Terapii Manualnej i Igłoterapii Suchej (ATMIS), 34-400 Nowy Targ, Poland; mateusz.kobylarz.fizjoterapia@gmail.com; 3 Faculty of Health Sciences, Universidad Francisco de Vitoria, 28223 Madrid, Spain; monica.lopezredondo@ufv.es (M.L.-R.); davinia.vicente@ufv.es (D.V.-C.); j.buffet.prof@ufv.es (J.B.-G.); 4 Department of Physiotherapy, Faculty of Nursery, Physiotherapy and Podiatry, Complutense University of Madrid, 28040 Madrid, Spain; juavaler@ucm.es

**Keywords:** inter-examiner agreement, minimal detectable change, muscle stiffness, piriformis muscle, reliability, shear wave elastography, ultrasound imaging

## Abstract

**Background/Objectives**: Shear wave elastography (SWE) may provide an objective method for quantifying piriformis muscle stiffness, but its clinical and research use requires evidence that the measurement procedure is reliable. This study aimed to determine the intra- and inter-examiner reliability of a standardized SWE protocol for assessing piriformis muscle stiffness and to provide measurement error thresholds for clinical interpretation. **Methods**: Twenty-one healthy volunteers were assessed bilaterally by two examiners with different levels of ultrasound experience. The piriformis muscle was identified in the long axis beneath the gluteus maximus, and SWE images were acquired using a standardized protocol. Each side was measured twice by each examiner, resulting in 168 ultrasound images. Reliability was analyzed using side-specific observations (*n* = 42). Intraclass correlation coefficients (ICCs), standard errors of measurement (SEMs) and minimal detectable changes (MDCs) were calculated for shear modulus and shear wave speed. **Results**: Inter-examiner reliability was good for single measurements, with ICCs of 0.872 for shear modulus and 0.813 for shear wave speed. When the average of two measurements was used, ICCs were similar, reaching 0.876 and 0.832, respectively. Inter-examiner MDC values ranged from 6.1 to 6.2 kPa for shear modulus and from 0.37 to 0.39 m/s for shear wave speed. Intra-examiner reliability was excellent for both examiners, with ICCs ranging from 0.938 to 0.979. Test–retest MDC values ranged from 2.7 to 3.3 kPa for shear modulus and from 0.19 to 0.22 m/s for shear wave speed. **Conclusions**: SWE provides good inter-examiner and excellent intra-examiner reliability for assessing piriformis muscle stiffness using a standardized acquisition protocol. Longitudinal assessments should preferably be performed by the same examiner, and changes should be interpreted in relation to SEM and MDC values, particularly in multi-examiner settings where absolute measurement error is larger. These thresholds reflect measurement error and should not be interpreted as evidence of diagnostic validity or responsiveness to treatment.

## 1. Introduction

The piriformis muscle is a deep external rotator of the hip located within the gluteal region. It originates from the anterior surface of the sacrum, passes laterally through the greater sciatic foramen, and inserts onto the superior aspect of the greater trochanter. Functionally, the piriformis contributes to hip external rotation when the hip is extended and to hip abduction when the hip is flexed [1]. Its clinical relevance is largely explained by its location within the deep gluteal space and its close anatomical relationship with the sciatic nerve and other neurovascular structures. In the most common anatomical pattern, the sciatic nerve exits the pelvis inferior to the piriformis muscle; however, anatomical variants are not rare. A recent systematic review and meta-analysis reported that the typical pattern is present in approximately 90% of cases, while in more than 10% of the population, the sciatic nerve courses through or above the piriformis muscle [2]. These anatomical relationships are important not only for diagnostic reasoning but also for the safety and precision of invasive and non-invasive procedures targeting the deep gluteal region [3].

Because of this close anatomical relationship, the piriformis muscle has been widely discussed in relation to buttock pain, sciatica-like symptoms, and non-discogenic sciatic nerve entrapment. Piriformis syndrome has traditionally been described as a form of sciatica caused by compression or irritation of the sciatic nerve by the piriformis muscle and is estimated to account for approximately 6–8% of sciatica cases [1]. Nevertheless, its diagnosis remains controversial because symptoms overlap with lumbar radiculopathy, lumbar spinal stenosis, sacroiliac joint pain, hip disorders, greater trochanteric pain syndrome, and other sources of posterior pelvic or gluteal pain [1,2]. For this reason, the broader term deep gluteal syndrome has been proposed to describe non-discogenic sciatic nerve entrapment in the subgluteal space, recognizing that structures other than the piriformis (such as fibrous bands, the obturator internus/gemelli complex, quadratus femoris, hamstring structures, or other gluteal tissues) may contribute to symptoms [2].

Although piriformis syndrome is commonly framed as a nerve entrapment condition, piriformis muscle dysfunction can also be understood within the broader context of myofascial pain. Myofascial pain is a soft-tissue pain syndrome characterized by local and referred musculoskeletal pain arising from myofascial trigger points (MTrPs), which are hyperirritable areas located within taut bands of skeletal muscle that may reproduce local or referred pain when stimulated [4]. In patients with low back pain, MTrPs are commonly identified in lumbar and gluteal muscles, including the quadratus lumborum, gluteus medius, gluteus minimus, psoas, iliocostalis lumborum, and piriformis [5,6]. A recent systematic review reported that active MTrPs in patients with low back pain may be present in the piriformis muscle in approximately 42% of cases, while latent MTrPs may also be found in symptomatic patients [6]. These findings support the clinical relevance of assessing the piriformis not only as a potential mechanical contributor to sciatic nerve irritation, but also as a possible source of local and referred myofascial pain.

From a pathophysiological perspective, MTrPs have been associated with altered local muscle physiology, including taut bands, local ischemia, hypoxia, pH reduction, and the release of inflammatory and nociceptive mediators such as bradykinin, serotonin, substance P, and calcitonin gene-related peptide [7,8]. Clinically, these changes may manifest as pain on compression, referred pain, increased resting tone, restricted mobility, altered motor control, and reduced tolerance to sustained or repeated loading [9]. In the piriformis muscle, this is particularly relevant because increased tone, shortening, hypertrophy, spasm, or taut painful bands may theoretically increase mechanical stress within the deep gluteal space and contribute to irritation of adjacent neural structures [10]. Therefore, quantifying the mechanical properties of the piriformis muscle could provide useful information for both clinical assessment and research, especially in conditions where muscle stiffness, shortening, or increased resting tone are considered part of the clinical presentation.

However, the clinical assessment of piriformis muscle tone and MTrPs remains challenging. Manual palpation is frequently used to identify taut bands, local tenderness, and symptom reproduction, but this approach is inherently examiner-dependent and may be influenced by the depth of the muscle, the thickness of the overlying gluteal tissues, the patient’s pain sensitivity, and the examiner’s experience [11,12,13]. In addition, the deep location of the piriformis makes direct palpation less precise than that in superficial muscles [11,12,13]. Imaging techniques may help overcome some of these limitations. For instance, magnetic resonance imaging can contribute to the assessment of deep gluteal syndrome by identifying anatomical abnormalities, muscle asymmetry, or sciatic nerve signal changes, but cost, availability, the controversial association between clinical complaints and radiological findings in some musculoskeletal conditions, and the lack of a single definitive diagnostic test limit their routine use [14,15]. Conventional ultrasound may help visualize the piriformis muscle and adjacent structures, but B-mode imaging alone provides limited information about tissue mechanical properties [16,17].

Shear wave elastography (SWE) is an ultrasound-based imaging technique that provides a quantitative estimate of tissue stiffness by measuring shear wave propagation through biological tissues [18]. In musculoskeletal research, SWE has increasingly been used to assess muscle stiffness in vivo because it is non-invasive, relatively accessible, and can provide quantitative outcomes such as shear wave speed or shear modulus [19]. Previous studies have shown that SWE can provide reliable assessments of skeletal muscle stiffness when acquisition conditions are carefully standardized, including participant position, probe orientation, muscle relaxation, transducer pressure, anatomical landmarking, and region-of-interest placement [20,21,22].

Emerging evidence suggests that SWE may be clinically useful for the assessment of piriformis muscle stiffness. Gülsaran et al. [23] evaluated patients with unilateral piriformis syndrome and reported significantly higher stiffness values on the pathological side than on the unaffected side, suggesting that SWE may provide additional diagnostic information when combined with conventional ultrasonography. Similarly, a case–control study including 40 patients with piriformis muscle syndrome found that piriformis muscle thickness and Young’s modulus were significantly higher on the symptomatic side than in healthy controls, with two-dimensional ultrasound combined with SWE showing promising diagnostic performance [24]. SWE has also been used to evaluate piriformis muscle elongation during stretching positions, supporting its ability to detect changes in piriformis mechanical behavior under different hip positions [25].

Despite these promising applications, clinical interpretation of piriformis stiffness values requires previous evidence that the measurement procedure itself is reliable. This is particularly important because US-based imaging is operator-dependent, and the piriformis muscle poses specific technical challenges due to its depth, oblique fiber orientation and anatomical variability. Reliability studies are therefore needed before SWE-derived piriformis stiffness can be confidently used to compare symptomatic and asymptomatic individuals, monitor treatment effects, evaluate changes after stretching or manual therapy, or establish reference values. In this context, intra-examiner reliability determines whether the same examiner can obtain stable measurements across repeated acquisitions, whereas inter-examiner reliability determines whether different examiners can obtain comparable values using the same standardized protocol. Error metrics such as the standard errors of measurement and minimal detectable change are also essential to determine whether observed changes exceed measurement noise.

Therefore, the aim of this study was to determine the intra- and inter-examiner reliability of piriformis muscle stiffness assessment using SWE under a standardized acquisition protocol by quantifying absolute reliability indices, including intraclass correlation coefficients, standard error of measurements and minimal detectable changes, in order to provide clinically interpretable benchmarks for future studies evaluating piriformis muscle dysfunction, myofascial pain, and piriformis syndrome.

## 2. Materials and Methods

### 2.1. Study Design

A longitudinal observational study was carried out between February and May 2026 to assess the inter-examiner reliability of an SWE protocol. Data collection was performed in a dedicated ultrasound laboratory located at the Faculty of Health Sciences of Francisco de Vitoria University, Spain. The study report was prepared in accordance with the recommendations of the Guidelines for Reporting Reliability and Agreement Studies (GRRAS) [26], and the EQUATOR Network recommendations [27] were followed. All procedures respected the ethical principles established in the Declaration of Helsinki. The study protocol was reviewed and approved by a local Ethics Committee before recruitment and data collection began.

### 2.2. Participants

Healthy volunteers were recruited through convenience sampling using notices displayed across the university campus. Participants were eligible if they were between 18 and 65 years old and reported no musculoskeletal pain either at the time of assessment or during the preceding six months. Individuals were excluded if they had received any medical, pharmacological, or physiotherapy treatment during the previous six months that could influence pain sensitivity or muscle tone. Additional exclusion criteria included previous traumatic injuries, a history of surgery, clinical signs or a previous diagnosis of radiculopathy or myelopathy, and the presence of systemic disease.

Volunteers who met the eligibility criteria were invited to read the study information carefully and provide written informed consent before being scheduled for the assessment sessions.

### 2.3. Sample Size Calculation

The required sample size was calculated according to the recommendations proposed by Walter et al. [28] for reliability studies based on intraclass correlation coefficients (ICCs). As no previous research had examined intra- or inter-examiner reliability for this specific procedure, the calculation was based on an a priori assumption. A minimum acceptable ICC value (ρ0) of 0.70 was selected, corresponding to the lower threshold for “good reliability” reported in the literature [29]. The anticipated ICC (ρ1) was set at 0.90, based on previous SWE reliability studies conducted in other muscles [30]. Considering a significance level of 0.05, a statistical power of 80%, two examiners, and an estimated attrition rate of 10% due to the longitudinal design, a total of 26 datapoints were required to ensure adequate statistical power.

This calculation referred to the minimum number of measurement units required for the reliability analysis. Because the protocol was designed as a bilateral assessment and the primary analysis was prespecified at the side-specific level, each piriformis muscle was considered a measurement unit. However, side-specific observations should not be interpreted as fully independent participants because measurements from the left and right sides of the same individual may be correlated. This issue was therefore considered when interpreting the precision and generalizability of the reliability estimates.

### 2.4. Examiners

Two examiners participated in the study. One was an experienced clinician with more than 10 years of experience in musculoskeletal ultrasound and in the clinical management of patients with musculoskeletal disorders. The second examiner was considered a novice, with less than one year of experience in both musculoskeletal ultrasound and clinical practice, and had completed 20 h of ultrasound training.

Before the beginning of the study, both examiners agreed on a standardized image acquisition protocol, as described in the Procedures section. This preliminary agreement included participant positioning, transducer placement, anatomical references to be used during image acquisition, and the criteria for defining the region of interest (ROI) when calculating the ultrasound-derived metrics. In particular, the examiners agreed on the anatomical boundaries to be considered during image analysis and on the methodological criteria for delimiting the area from which the measurements would be extracted.

The order of participants, the examiner assigned to each assessment session, and the initial side evaluated were randomized. Data collection was organized into two independent time blocks (a morning session from 9:00 to 11:00 and an afternoon session from 13:00 to 15:00). To avoid communication, consensus, or the exchange of information between examiners, a strict isolation procedure was applied, and each examiner performed the assessments on alternating days.

Each participant attended two separate assessment sessions, scheduled 24 h apart. The examiner responsible for image acquisition was alternated between sessions. After data collection, a third investigator anonymized and coded all ultrasound images. Subsequently, each examiner measured their own images in a randomized order while remaining blinded to participant identity and the side assessed.

### 2.5. Shear Wave Elastography Protocol

Ultrasound imaging was performed with a Canon Aplio A system and an 8C1 convex probe (Canon Medical Corp, Otawara, Japan). The same preset parameters were maintained throughout the study for all acquisitions, including a frequency of 5 MHz, a gain of 80 dB, a dynamic range of 60, and an imaging depth of 10 cm.

The ultrasound procedure used to identify the piriformis muscle was based on the approach described by Siahaan et al. [31] to calculate piriformis muscle thickness in its longitudinal axis.

**Figure 1:**
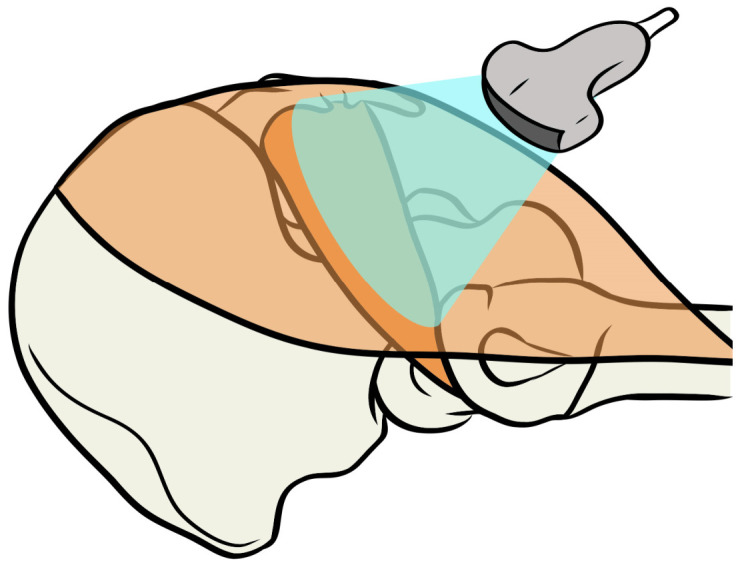
Illustration showing the transducer placement for ultrasound examination of the piriformis muscle. The probe is positioned over the gluteal region to obtain a long-axis view of the piriformis muscle, located deep to the gluteus maximus.

Participants were positioned in prone lying with the lower limbs relaxed to minimize muscle activation and avoid changes in muscle morphologym and prevent morphological bias attributable to muscle contractions [32]. Before placing the transducer, the main anatomical landmarks (the posterior superior iliac spine, the sacral hiatus and the greater trochanter) were identified by palpation. The probe was initially positioned over the line extending from the midpoint between the posterior superior iliac spine and the sacral hiatus toward the greater trochanter. From this starting position, the transducer was slowly moved caudally and slightly adjusted in orientation until a clear long-axis image of the piriformis muscle was obtained (Figure 1). Examiners were instructed to apply minimal pressure and avoid visible soft-tissue deformation; however, for pragmatic reasons, probe pressure was not objectively quantified (although this could be interpreted as a methodological flaw, this decision reflects routine clinical practice where external pressure-monitoring devices are not usually used, and was consistent with the pragmatic aim of developing a transferable assessment procedure).

During scanning, the examiner aimed to identify the piriformis as a deep muscular structure located beneath the gluteus maximus, following an oblique trajectory from the sacral region toward the greater trochanter. The sciatic nerve (although not assessed in this study) was identified in relation to the deep/inferior aspect of the piriformis muscle, appearing as a rounded or oval hyperechoic structure with a fascicular pattern.

**Figure 2:**
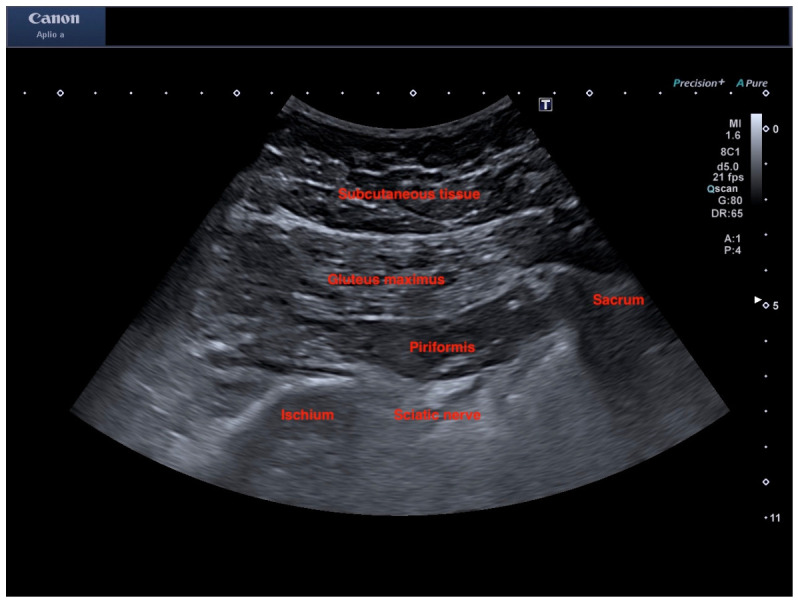
Illustrative example of a long-axis view of the piriformis muscle acquired with B-mode ultrasound.

The probe was carefully tilted and adjusted to obtain the clearest possible visualization of the piriformis muscle, with the ultrasound beam oriented as perpendicular as possible to the superficial and deep muscle borders (to reduce angle-dependent artifacts such as anisotropy and to improve the definition of the fascial limits used for thickness measurement). The image was considered acceptable when the superficial and deep borders of the piriformis muscle were clearly visible and the sciatic nerve could be identified in the same field of view. Care was taken to apply minimal transducer pressure to avoid deformation of the soft tissues and to maintain a consistent probe orientation across participants (Figure 2).

Once the longitudinal B-mode image of the piriformis muscle had been optimized, the SWE mode was activated. The elastography sampling box was positioned over the central portion of the piriformis muscle and adjusted according to each participant’s anatomy, ensuring that the target area was adequately covered while avoiding unnecessary inclusion of adjacent tissues. The transducer was kept as stable as possible, and the ultrasound beam was maintained as perpendicular as possible to the muscle borders to reduce angle-dependent artifacts and improve the reliability of the elastographic signal.

**Figure 3:**
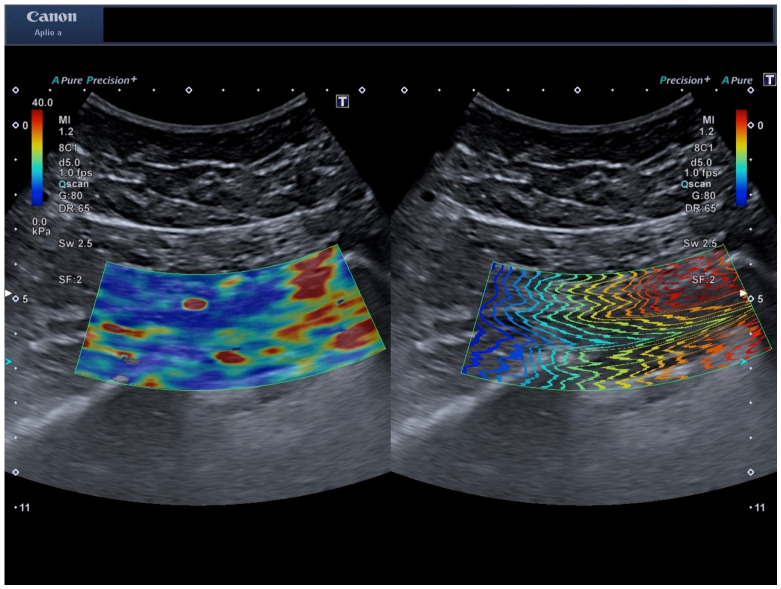
Illustrative example of a long-axis view of the piriformis muscle acquired with shear wave elastography and elastography sampling boxes.

After activating SWE, the examiner waited for the elastographic image to stabilize for approximately 10–15 s before recording the acquisition. The elastography sampling box was not standardized to a fixed millimetric dimension but was adapted to each participant’s anatomy in order to cover the maximum portion of the piriformis muscle while avoiding unnecessary inclusion of adjacent tissues. This approach was selected because the piriformis is a deep and obliquely oriented muscle, and the visible muscle area may vary according to individual anatomy, muscle depth, and overlying gluteal tissue thickness. Images were only stored when the qualitative appearance of the SWE box showed adequate signal filling, without visible signal dropouts, transparent gaps, or areas suggesting poor shear wave propagation. If the elastogram showed an unstable color distribution or incomplete filling of the sampling box, the probe position was readjusted and the acquisition was repeated until an acceptable image was obtained (Figure 3). Each SWE acquisition corresponded to one stored image, and no frame-by-frame temporal averaging was performed.

All SWE images were first acquired and stored without performing measurements during the scanning session. After image acquisition, the stored images were anonymized and later assessed offline in a randomized order. For measurement, the ROI was not defined as a fixed circular or rectangular area. Instead, a freely drawn quantification ROI was placed within the visible limits of the piriformis muscle. This approach allowed the examiner to adapt the measurement area to the visible morphology of the muscle belly while minimizing contamination from adjacent tissues. To reduce operator discretion, ROI placement followed predefined contouring criteria: the ROI had to remain within the visible limits of the piriformis muscle, avoid contact with the superficial and deep muscle borders, and exclude the surrounding fasciae, sciatic nerve, gluteal tissue, and other adjacent perimuscular structures. This approach was intended to ensure that stiffness values were obtained from the muscle belly itself rather than from fascial, neural, or perimuscular tissues. The ultrasound system automatically provided shear wave speed values and converted these data into shear modulus values using the built-in software of the Canon Aplio A system. No external or manual post-processing was applied after image acquisition.

Shear wave speed and shear modulus were recorded. While shear wave speed is the direct physical output of SWE, shear modulus is a device-derived estimate calculated by the ultrasound system. This conversion assumes simplified tissue behavior, including homogeneity, isotropy, linear elasticity, incompressibility, and constant tissue density. These assumptions are imperfect for skeletal muscle because muscle is anisotropic, viscoelastic, and direction-dependent, especially when the target muscle is deep and obliquely oriented [33,34]. Therefore, shear wave speed should be regarded as the primary SWE outcome in the present study, while shear modulus values should be interpreted as secondary system-derived estimates reported for clinical readability and comparison with previous piriformis SWE studies.

### 2.6. Statistical Analysis

All statistical analyses were conducted using the Statistical Package for the Social Sciences software (SPSS), version 31 for Mac OS (Armonk, NY, USA). Statistical significance was set at *p* < 0.05, and all analyses were performed using two-tailed tests. The normality of continuous variables was assessed visually through histogram inspection and analytically using the Shapiro–Wilk test.

Demographic and sonographic characteristics of the participants were reported using descriptive statistics. Categorical data (i.e., gender) were expressed as absolute frequencies and percentages. Continuous variables (i.e., age, height, weight, body mass index and SWE metrics) were reported as means and standard deviations after verifying a normal distribution. Sex differences were explored using independent-samples Student’s *t*-tests, with mean differences, 95% confidence intervals, and *p*-values reported.

Reliability analyses were performed for the elastographic variables obtained from the piriformis muscle. For each parameter, the following outcomes were calculated: (1) the mean and standard deviation of the values obtained by each examiner; (2) the disagreement between measurements, calculated as the difference between repeated trials for intra-examiner reliability and as the difference between examiners for inter-examiner reliability, considering both single measurements and the average of two measurements (using *t*-tests and reporting the mean difference, 95% confidence interval and *p* value); (3) the absolute error, to avoid cancelation of positive and negative differences and to provide a direct estimate of measurement variability; (4) intraclass correlation coefficients using a two-way mixed-effects model for consistency, applying ICC_3,1_ for single-measurement reliability and ICC_3,2_ for the reliability using the mean average of two measurements [29]; (5) the standard error of measurement (SEM), calculated as the standard deviation of the mean multiplied by the square root of 1 minus the ICC; and (6) the minimal detectable change (MDC), calculated as 1.96 × √2 × SEM [35]. A two-way mixed-effects model was selected because the two examiners were not randomly sampled from a broader population of possible raters but were deliberately selected to represent two predefined levels of ultrasound experience. Therefore, participants/side-specific measurement units were treated as random, whereas examiners were treated as fixed. Accordingly, the inter-examiner ICCs should be interpreted as estimates of reliability between these two examiner profiles under the standardized protocol used in the present study, rather than as estimates intended to generalize to all possible examiners.

The interpretation of ICC values followed the thresholds proposed by Koo et al. [29]. Values below 0.50 were interpreted as poor reliability, values between 0.50 and 0.75 as moderate reliability, values between 0.75 and 0.90 as good reliability, and values above 0.90 as excellent reliability.

## 3. Results

During recruitment, 21 volunteers expressed interest in participating in the study. All volunteers met the eligibility criteria, and none were excluded at this stage. During the study procedures, no participants were excluded or lost to follow-up; therefore, the final sample included 21 participants, all of whom were included in the analyses. Regarding the number of data points, considering that both sides were assessed, each measurement was performed twice, and assessments were conducted by two examiners, a total of 168 ultrasound images were acquired. Therefore, considering that both sides were assessed, the unit of analysis for reliability was the side-specific measurement, resulting in 42 observations (21 participants × 2 sides). Inter-examiner agreement based on individual measurements was therefore analyzed using *n* = 42 paired observations. When the average of the two repeated measurements was used, the sample size remained *n* = 42, as the two trials were averaged to obtain a single value for each side and examiner. Intra-examiner reliability was also analyzed using *n* = 42 paired observations, corresponding to the repeated measurements obtained for each side.

Participants’ characteristics are summarized in Table 1. Gender comparisons revealed that males and females were comparable in age, with no statistically significant difference between groups (*p* = 0.567). However, significant sex-related differences were observed for the remaining demographic variables. Males showed greater height and body weight than females (both *p* < 0.001), as well as a significantly higher BMI (*p* = 0.012). In contrast, shear wave elastography outcomes were higher in females than in males. Females demonstrated significantly greater shear modulus and shear wave speed values, with both comparisons reaching statistical significance (*p* < 0.001). It should be noted that the sample size of this study was calculated for the reliability analysis and not for the establishment of normative reference values or the assessment of sex-related differences. Therefore, these comparisons are presented for descriptive purposes only and should not be considered inferential or clinically relevant findings of the study.

Inter-examiner reliability results for piriformis muscle stiffness are shown in Table 2. For single measurements, no significant systematic differences (*p* > 0.05) were observed between the experienced and novice examiners for either shear modulus or shear wave speed. The mean between-examiner difference was small for shear modulus, and ICC values indicated good inter-examiner relative reliability. However, the absolute magnitude of measurement error should also be considered, as the SEM represented 12.4% of the mean value and the MDC represented 33.3%. For shear wave speed, the between-examiner difference was also small and non-significant (*p* > 0.05), and ICCs indicated good inter-examiner reliability, although slightly lower than those obtained for shear modulus. In relative terms, the SEM and MDC for shear wave speed represented 6.0% and 16.8% of the mean value, respectively.

When the mean of two measurements was used, the reliability estimates remained stable. For shear modulus, the between-examiner difference was negligible and non-significant (*p* > 0.05), while the absolute error remained comparable to that observed for single measurements. The SEM and MDC represented 11.8% and 32.8% of the mean shear modulus value, respectively. For shear wave speed, averaging two measurements slightly improved the ICC and reduced the MDC, with SEM and MDC values corresponding to 6.0% and 15.9% of the mean value, respectively.

Overall, these findings indicate that SWE provides good inter-examiner relative reliability for assessing piriformis muscle stiffness, even when measurements are performed by examiners with different levels of experience. However, the absolute inter-examiner error, particularly for device-derived shear modulus, is not trivial. Therefore, small differences between examiners should be interpreted cautiously, and changes observed in multi-examiner settings should be interpreted in relation to the larger inter-examiner MDC thresholds. Averaging two measurements produced only minor improvements in reliability and measurement error, suggesting that single measurements may provide acceptable relative reliability, although averaged values may still be preferable when greater precision is required.

Test–retest reliability results for piriformis muscle stiffness are displayed in Table 3. For the experienced examiner, no significant systematic differences were observed between trials for either shear modulus or shear wave speed (*p* > 0.05). The mean differences were small, and the absolute errors were low for both outcomes. ICCs indicated excellent reliability for both shear modulus and shear wave speed. The SEM values were 0.95 kPa for shear modulus and 0.08 m/s for shear wave speed, corresponding to 5.1% and 3.5% of the respective mean values. The MDC values were 2.7 kPa and 0.22 m/s, corresponding to 14.5% and 9.5% of the respective mean values. These results indicate that repeated measurements performed by the experienced examiner showed limited test–retest measurement error, although changes smaller than these MDC thresholds should not be interpreted as true changes beyond measurement variability.

For the novice examiner, test–retest reliability also showed no significant systematic differences between trials for either shear modulus or shear wave speed (*p* > 0.05). The absolute errors were slightly higher for shear modulus than those observed for the experienced examiner, whereas the absolute error for shear wave speed was comparable. ICCs indicated excellent reliability for both shear modulus and shear wave speed. The SEM values were 1.18 kPa for shear modulus and 0.06 m/s for shear wave speed, corresponding to 6.3% and 2.6% of the respective mean values. The MDC values were 3.3 kPa and 0.19 m/s, corresponding to 17.7% and 8.1% of the respective mean values. Therefore, although the repeatability of the measurements performed by the novice examiner was also high, the relative MDC values indicate that only changes exceeding these thresholds should be considered beyond expected test–retest error.

Overall, these findings indicate that, as expected, intra-examiner metrics were superior to those for inter-examiner reliability. Shear wave elastography provides excellent test–retest reliability for assessing general piriformis muscle stiffness, regardless of examiner experience. The small between-trial differences, low absolute errors, and narrow MDC values suggest that repeated measurements are stable over time and that changes exceeding approximately 2.7–3.3 kPa for shear modulus or 0.19–0.22 m/s for shear wave speed may be interpreted as real changes beyond measurement error.

## 4. Discussion

The main strength of the present study is that it provides measurement error thresholds that may help interpret piriformis muscle stiffness values obtained with SWE. Although these values may not be fully extrapolated to other populations because demographic, anthropometric, and clinical characteristics can differ substantially across studies (and consequently impact muscle stiffness [36]), they provide a useful reference to determine whether observed differences are likely to represent true changes or may simply reflect measurement variability. This is particularly relevant in two common scenarios. First, when comparing stiffness values between groups, such as patients and controls, and second, when interpreting changes over time (for example, after treatment [37,38], after stretching [39], or when measurements are performed under different positions or tasks [40]). In these contexts, the ICC indicates the degree of relative reliability, but the SEM and MDC are especially informative because they indicate the magnitude of change required to exceed measurement error (but these thresholds should be interpreted only as measurement error references and not as evidence of diagnostic validity, clinical utility, or responsiveness to treatment). The absolute magnitude of measurement error should also be considered in relation to the mean stiffness values. Although inter-examiner ICCs indicated good relative reliability, the inter-examiner MDC for shear modulus represented approximately one-third of the mean value (32.8–33.3%). Therefore, the absolute inter-examiner error for device-derived shear modulus is not trivial. The relative MDC was lower for shear wave speed, approximately 15.9–16.8%, supporting its interpretation as the primary SWE outcome. In contrast, intra-examiner MDC percentages were smaller, ranging from approximately 14.5% to 17.7% for shear modulus and from approximately 8.1% to 9.5% for shear wave speed. These findings reinforce that longitudinal assessments should preferably be performed by the same examiner and that changes observed in multi-examiner settings should be interpreted using the larger inter-examiner MDC thresholds.

This is important when interpreting previous case–control findings in patients with piriformis muscle syndrome. Xu et al. [24] reported that piriformis muscle thickness and Young’s modulus were significantly higher on the affected side of patients with piriformis muscle syndrome than in controls, and they also found a moderate positive correlation between piriformis muscle thickness and Young’s modulus. In their study, the mean Young’s modulus in patients was approximately 19 kPa, whereas control values were approximately 9 kPa, suggesting a between-group difference of around 10 kPa. This difference is greater than both the test–retest MDC observed in the present study and the inter-examiner MDC obtained using either a single measurement or the average of two measurements. Therefore, although direct comparison between studies should be made cautiously because of differences in sample characteristics, clinical status, equipment, and acquisition protocols, the magnitude of the difference reported by Xu et al. [24] is likely to exceed measurement error and may reflect a real increase in piriformis muscle stiffness in symptomatic individuals.

These findings are also clinically relevant because piriformis muscle syndrome remains difficult to diagnose, with no universally accepted criterion standard. Xu et al. [24] emphasized that clinical diagnosis is often based on subjective tests and exclusion criteria, whereas imaging techniques such as MRI may be useful but are limited by time, cost, and accessibility. In this context, SWE may provide an objective and quantitative complement to clinical assessment (although its diagnostic validity and clinical responsiveness for piriformis-related pain conditions remain to be established). However, the interpretation of SWE findings should not rely only on statistical significance or diagnostic cut-off values. The present reliability data suggest that, for SWE to be clinically meaningful, observed differences should also be interpreted in relation to the appropriate MDC threshold. For example, when different examiners are involved, differences smaller than approximately 6.1–6.2 kPa for shear modulus or 0.37–0.39 m/s for shear wave speed should be interpreted cautiously, whereas larger differences are more likely to represent true stiffness differences rather than examiner-related variability.

Previous research has also examined the reliability of B-mode ultrasound measurements of the piriformis muscle. Çaglar and Ozyemisci-Taskiran [41] evaluated the interrater reliability of B-mode ultrasound measurements of piriformis muscle thickness in patients with piriformis syndrome. In their study, two experienced physiatrists measured piriformis muscle thickness at three anatomical regions, and the authors reported good interrater reliability, with an overall ICC of 0.836 and regional ICC values ranging from 0.777 to 0.883. These findings support the feasibility of using B-mode ultrasound to obtain reliable morphological measurements of the piriformis muscle.

However, those findings should be considered complementary to the present results rather than directly comparable. Conventional B-mode ultrasound provides structural information (e.g., muscle thickness, perimeter, cross-sectional area, shape or brightness), whereas SWE provides quantitative information about tissue stiffness. These outcomes may be related in some clinical contexts, but they are not interchangeable. Muscle thickness reflects morphology, while SWE-derived stiffness may reflect changes in muscle mechanical behavior, tone, contraction state, passive tension, or tissue composition.

Therefore, the present study extends the existing evidence in several ways. First, it specifically evaluates the reproducibility of SWE-derived piriformis muscle stiffness rather than B-mode thickness. Second, it provides both intra- and inter-examiner reliability estimates, whereas previous piriformis ultrasound reliability research has mainly focused on interrater agreement. Third, it includes absolute measurement error indices, including SEM and MDC values, which allow clinicians and researchers to determine whether observed differences or longitudinal changes are likely to exceed measurement variability. Finally, reliability was tested between examiners with different levels of ultrasound experience, which may better reflect the variability expected when SWE protocols are implemented in clinical or research settings.

The present results are also useful for interpreting studies that assess piriformis muscle stiffness under different biomechanical conditions. In the study conducted by Itsuda et al. [25], SWE was used to determine which hip position produced the greatest piriformis muscle elongation. The authors reported that the shear elastic modulus was higher with greater hip adduction and was higher during external rotation than internal rotation, with the greatest values observed in maximum external rotation combined with 110° hip flexion and 40° hip adduction. These findings support the use of SWE to detect position-dependent changes in piriformis muscle mechanical properties.

However, the interpretation of these position-related changes should also consider measurement error. Since this type of design usually involves repeated measurements under different conditions within the same participant, the test–retest MDC values from the present study may be the most appropriate reference when the same examiner performs all acquisitions. In that situation, changes exceeding approximately 2.7–3.3 kPa for shear modulus or 0.19–0.22 m/s for shear wave speed may be interpreted as changes beyond expected repeated-measurement error. Conversely, if different examiners acquire measurements across conditions or time points, the larger inter-examiner MDC values should be applied. This distinction is relevant because a change that may be considered real in a same-examiner longitudinal design may not be large enough to exceed the error expected when different examiners are involved.

Finally, these comparisons also highlight the importance of population-specific interpretation. The stretching-position study was conducted in healthy young men and excluded several participants because the piriformis muscle could not be measured, with body mass and BMI differing between measurable and non-measurable participants. This supports the idea that factors such as body composition, muscle depth, sex, age, and clinical status may influence the feasibility and interpretation of SWE measurements. Therefore, the MDC values reported in the present study should not be interpreted as universal thresholds but rather as reference values obtained under a specific protocol and sample. Even so, they provide a practical framework for deciding whether differences between groups, changes after treatment, or changes induced by stretching or hip positioning are likely to be real or attributable to measurement error.

The role of operator experience should also be considered when interpreting the present findings. Piriformis SWE is technically demanding because the muscle is located deep within the gluteal region and has an oblique orientation. Therefore, small variations in anatomical landmarking, probe angulation, transducer pressure, anisotropy control, elastogram stabilization, and ROI placement may influence the measured stiffness values. This issue is not exclusive to the piriformis muscle. Previous ultrasound research has shown that image interpretation may be affected by evaluator experience, particularly when fine anatomical structures are assessed [42]. However, evidence from studies evaluating superficial nerve fascicles with B-mode or high-resolution ultrasound should be extrapolated cautiously to the present context because SWE of a deep muscle using a convex probe involves different acquisition constraints and sources of variability.

Evidence from SWE studies is more directly relevant to the present study. Hudson et al. [43] reported that SWE reliability in the liver was influenced by the operator and by probe repositioning, with intra-operator reliability generally exceeding inter-operator reliability. Similarly, phantom studies have shown that SWE measurements may vary according to operator, equipment, probe characteristics, acquisition depth, target size, and ROI selection [44,45]. These findings support the need for careful standardization of SWE procedures, particularly when the target structure is deep or when measurements are performed by different examiners. In addition, reliability studies in musculoskeletal tissues have also emphasized that SWE measurements may be influenced by anatomical region, tissue characteristics, and acquisition protocol, further supporting the need for protocol-specific interpretation [46].

In the present study, several methodological precautions were adopted to reduce operator-dependent variability. Before data collection, both examiners agreed on a standardized protocol including participant positioning, anatomical references, transducer placement, acquisition criteria, and ROI delimitation. In addition, the novice examiner completed 20 h of ultrasound training before the study. The protocol also required minimal transducer pressure, careful adjustment of probe orientation, stabilization of the elastographic signal before image storage, and exclusion of images with poor filling, signal dropout, or unstable color distribution.

The comparison between examiners with different levels of experience provides clinically relevant information. Although one examiner had more than 10 years of musculoskeletal ultrasound experience and the other had less than one year of experience, no significant systematic differences were observed between examiners, and inter-examiner reliability was good for both shear modulus and shear wave speed. This suggests that, when a standardized protocol and prior training are used, SWE assessment of piriformis stiffness may be feasible even when examiners differ in experience. Nevertheless, inter-examiner MDC values were larger than intra-examiner MDC values, indicating that examiner-related variability was not completely eliminated. Therefore, longitudinal assessments should preferably be performed by the same examiner, and multi-examiner studies should apply standardized training procedures and interpret changes using the larger inter-examiner MDC thresholds.

Based on our results, three practical recommendations can be provided for the assessment of piriformis muscle stiffness using SWE. First, longitudinal assessments should preferably be performed by the same examiner whenever possible. Although inter-examiner reliability was good, test–retest reliability showed lower measurement error than inter-examiner comparisons. In repeated assessments performed by the same examiner, the MDC ranged from 2.7 to 3.3 kPa for shear modulus and from 0.19 to 0.22 m/s for shear wave speed, depending on examiner experience. Therefore, changes exceeding these values may be interpreted as true changes beyond measurement error when the same examiner performs the follow-up evaluations.

Second, when more than one examiner is involved, the results suggest that SWE can still provide reliable measurements of piriformis muscle stiffness, even when examiners have different levels of experience. No significant systematic differences were observed between the experienced and novice examiners, and ICC values indicated good inter-examiner reliability. However, the inter-examiner MDC values were higher than the test–retest MDC values. For single measurements, the MDC was 6.2 kPa for shear modulus and 0.39 m/s for shear wave speed. When the average of two measurements was used, these values were only slightly reduced to 6.1 kPa and 0.37 m/s, respectively. This indicates that multi-examiner designs require larger changes to be considered real and not attributable to measurement variability.

Finally, the decision to average two measurements instead of using a single measurement should be based on a cost–benefit assessment. In the present study, averaging two measurements produced only minimal improvements in reliability and measurement error. For shear modulus, the inter-examiner MDC was almost unchanged when using the mean of two measurements instead of a single measurement, and for shear wave speed, the improvement was also small. Therefore, in routine clinical contexts or when time efficiency is important, a single measurement may be sufficient, provided that the acquisition procedure is standardized. However, averaging two measurements may still be justified in research settings, in longitudinal studies where small changes are expected, or in multi-examiner protocols where maximizing precision is particularly important.

### Limitations

The present study has several limitations that should be considered when interpreting the findings. First, the sample consisted of healthy volunteers recruited by convenience sampling, and most participants were young female adults. Therefore, the results may not be directly generalizable to older individuals, patients with piriformis syndrome, deep gluteal syndrome, low back pain, myofascial pain, or populations with greater variability in body composition and muscle depth, as these conditions may hinder clear visualization of the piriformis muscle and accurate delineation of the ROI. Second, the sample size was calculated to assess reliability and not to establish normative reference values or to explore subgroup differences according to sex, age, body mass index, or clinical status. Consequently, the descriptive differences observed between males and females should be interpreted cautiously and should not be considered clinically conclusive. Third, each side was considered a separate measurement unit for the primary reliability analysis. This decision was made a priori to preserve the side-specific nature of the ultrasound acquisition protocol, as each side required separate anatomical landmarking, transducer placement, SWE acquisition, image storage, and ROI placement. In addition, piriformis-related symptoms and stiffness differences may occur unilaterally; therefore, the side, rather than the participant, was considered the most clinically relevant unit for evaluating the repeatability of the procedure. However, these observations should not be interpreted as 42 fully independent participants because the left and right sides from the same individual showed moderate correlations (r ≈ 0.5). Alternative participant-level approaches need to be considered in future studies (i.e., analysis of one randomly selected side per participant, averaging both sides to obtain a single participant-level value, or applying mixed-effects/generalizability models with side nested within participant).

Another limitation is that reliability was evaluated using a single ultrasound system, one transducer, fixed preset parameters, and a specific acquisition protocol. Since SWE values may vary depending on the device, software, probe characteristics, acquisition settings, transducer pressure, region-of-interest placement, and participant positioning, the measurement error values reported in this study should be interpreted as protocol-specific rather than universal thresholds. In addition, only short-term test–retest reliability was assessed, with repeated measurements performed under controlled laboratory conditions. Therefore, further studies are needed to determine whether similar reliability can be achieved over longer follow-up periods, in less controlled clinical environments, or when measurements are performed by a larger number of examiners with different levels of training.

Finally, this study was designed to evaluate reliability and measurement error, but it did not assess diagnostic validity, responsiveness to treatment, or the association between piriformis stiffness and clinical symptoms. Although the sciatic nerve was identified during image acquisition to assist with anatomical orientation, its morphology or mechanical relationship with the piriformis muscle was not analyzed. Future studies should examine whether SWE-derived piriformis stiffness can discriminate between symptomatic and asymptomatic individuals, detect clinically meaningful changes after interventions, and contribute to the assessment of patients with suspected piriformis-related or deep gluteal pain conditions.

## 5. Conclusions

This study showed that piriformis muscle stiffness can be assessed reliably using SWE when a standardized acquisition protocol is applied. Inter-examiner reliability was good for both shear modulus and shear wave speed, even when measurements were performed by examiners with different levels of ultrasound experience. Intra-examiner reliability was excellent, with lower measurement error than that observed between examiners. These findings suggest that longitudinal evaluations should preferably be performed by the same examiner whenever possible.

The reported SEM and MDC values provide clinically interpretable thresholds for determining whether observed differences in piriformis muscle stiffness are likely to exceed measurement error. Changes greater than approximately 2.7–3.3 kPa or 0.19–0.22 m/s may be interpreted as real changes when the same examiner performs repeated assessments, whereas larger thresholds of approximately 6.1–6.2 kPa or 0.37–0.39 m/s should be considered when different examiners are involved. Averaging two repeated measurements produced only minor improvements in reliability, suggesting that a single standardized measurement may be acceptable in routine settings, although averaged values may be preferable in research contexts or when greater precision is required.

## Figures and Tables

**Table 1:** Descriptive analyses of demographic characteristics of the sample.

Variables	Males (*n* = 6)	Females (*n* = 15)	Gender Difference (95% CI)
Demographics			
Age, years	20.6 ± 1.2	20.3 ± 1.2	0.3 (−0.8; 1.5) *p* = 0.567
Height, m	1.76 ± 0.05	1.64 ± 0.04	0.12 (0.07; 0.16) *p* < 0.001
Weight, kg	75.0 ± 6.4	58.5 ± 6.6	16.5 (9.9; 23.2) *p* < 0.001
BMI, kg/m^2^	24.0 ± 1.9	21.5 ± 1.8	2.5 (0.6; 4.4) *p* = 0.012
Shear wave elastography metrics *			
Shear modulus, kPa	15.9 ± 5.3	19.7 ± 6.3	−3.8 (−5.9; −1.8) *p* < 0.001
Shear wave speed, m/s	2.19 ± 0.35	2.38 ± 0.30	−0.18 (−0.29; −0.08) *p* < 0.001

* Elastography values correspond to a single pooled value calculated by averaging both measurements performed by both examiners.

**Table 2:** Inter-examiner reliability analyses to determine general piriformis muscle stiffness.

Reliability Estimates	Shear Modulus (kPa)		Shear Wave Speed (m/s)	
	Experienced Examiner	Novice Examiner	Experienced Examiner	Novice Examiner
Single Measurements				
Mean	18.5 ± 6.7	18.7 ± 5.9	2.29 ± 0.33	2.35 ± 0.32
Difference	−0.3 (−3.1; 2.6) *p* = 0.844		−0.07 (−0.20; 0.08) *p* = 0.418	
Absolute Error	3.4 ± 2.5		0.20 ± 0.16	
ICC_3,1_, 0–1	0.872 (0.763; 0.931)		0.813 (0.653; 0.900)	
SEM	2.3		0.14	
SEM (%)	12.4		6.0	
MDC	6.2		0.39	
MDC (%)	33.3		16.8	
Mean Average of 2 Measurements				
Mean	18.6 ± 6.6	18.6 ± 5.9	2.31 ± 0.33	2.34 ± 0.33
Difference	0.0 (−1.9; 1.9) *p* = 0.992		−0.02 (−0.12; 0.07) *p* = 0.643	
Absolute Error	3.4 ± 2.5		0.19 ± 0.15	
ICC_3,2_, 0–1	0.876 (0.808; 0.919)		0.832 (0.740; 0.891)	
SEM	2.2		0.14	
SEM (%)	11.8		6.0	
MDC	6.1		0.37	
MDC (%)	32.8		15.9	

ICC: Intraclass correlation coefficients; MDC: Minimal Detectable Changes; SEM: Standard Error of Measurement.

**Table 3:** Test–retest reliability analyses to determine general piriformis muscle stiffness.

Reliability Estimates	Experienced Examiner				Novice Examiner			
	Shear Modulus (kPa)		Shear Wave Speed (m/s)		Shear Modulus (kPa)		Shear Wave Speed (m/s)	
	Trial 1	Trial 2	Trial 1	Trial 2	Trial 1	Trial 2	Trial 1	Trial 2
Mean	18.5 ± 6.7	18.8 ± 6.7	2.29 ± 0.33	2.33 ± 0.32	18.7 ± 5.9	18.6 ± 6.0	2.35 ± 0.32	2.32 ± 0.34
Difference	−0.3 (−3.2; 2.6) *p* = 0.858		−0.04(−0.17; 0.11) *p* = 0.629		0.1 (−2.5; 2.7) *p* = 0.933		0.03 (−0.10; 0.18) *p* = 0.634	
Absolute Error	1.3 ± 1.4		0.11 ± 0.12		1.8 ± 1.4		0.11 ± 0.08	
ICC_3,1_, 0–1	0.979 (0.961; 0.989)		0.938 (0.884; 0.967)		0.960 (0.925; 0.978)		0.958 (0.923; 0.978)	
SEM	1.0		0.08		1.1		0.06	
SEM (%)	5.1		3.5		6.3		2.6	
MDC	2.7		0.22		3.3		0.19	
MDC (%)	14.5		9.5		17.7		8.1	

ICC: Intraclass correlation coefficients; MDC: Minimal Detectable Changes; SEM: Standard Error of Measurement.

## Data Availability

The datasets used and/or analyzed during the current study are available from the corresponding author on reasonable request.
